# An Adaptive Failure Detector Based on Quality of Service in Peer-to-Peer Networks

**DOI:** 10.3390/s140916617

**Published:** 2014-09-05

**Authors:** Jian Dong, Xiao Ren, Decheng Zuo, Hongwei Liu

**Affiliations:** School of Computer Science and Technology, Harbin Institute of Technology, Harbin 150001, China; E-Mails: renxiao@hit.edu.cn (X.R.); zuodc@hit.edu.cn (D.Z.); liuhw@hit.edu.cn (H.L.)

**Keywords:** distributed system, Peer-to-Peer networks, adaptive failure detector, quality of service

## Abstract

The failure detector is one of the fundamental components that maintain high availability of Peer-to-Peer (P2P) networks. Under different network conditions, the adaptive failure detector based on quality of service (QoS) can achieve the detection time and accuracy required by upper applications with lower detection overhead. In P2P systems, complexity of network and high churn lead to high message loss rate. To reduce the impact on detection accuracy, baseline detection strategy based on retransmission mechanism has been employed widely in many P2P applications; however, Chen's classic adaptive model cannot describe this kind of detection strategy. In order to provide an efficient service of failure detection in P2P systems, this paper establishes a novel QoS evaluation model for the baseline detection strategy. The relationship between the detection period and the QoS is discussed and on this basis, an adaptive failure detector (B-AFD) is proposed, which can meet the quantitative QoS metrics under changing network environment. Meanwhile, it is observed from the experimental analysis that B-AFD achieves better detection accuracy and time with lower detection overhead compared to the traditional baseline strategy and the adaptive detectors based on Chen's model. Moreover, B-AFD has better adaptability to P2P network.

## 1. Introduction

Recently, Peer-to-Peer (P2P) network has rapidly become the major computing platform to share resources and services on Internet [1,2]. It can greatly improve network efficiency by taking full advantage of network bandwidth and the computing power of individual network nodes. An important guarantee to maintain the efficiency of P2P network is the high availability in the presence of node failures, as a basic building block for a distributed system of high availability [3], failure detector has always been a hot research topic in the field of P2P networks.

In P2P networks, failure detector provides support for routing recovery and update when failure occurs to maintain the validity of system topology by periodically probing the states of nodes in the system. Quality of Service (QoS) provided by failure detector is an important factor that affects the performance of P2P system, poor accuracy of detection will lead to plenty of unnecessary routing repair and data transfer, which will greatly increase the system overhead, especially in structured P2P networks. The reliability of routing imposes higher requirements on detection time, and the loss of normal packets (sent to failed nodes) has a great impact on the performance of upper applications such as task completion time, network throughput, video frame loss rate, *etc.* [4]. Meanwhile, the large scale and high churn of P2P networks [5,6] generate heavy detection overhead, which have become the major source of P2P traffic [7]. In the early version of Gnutella, the number of PING messages used for failure detection has exceeded 50% of the entire traffic [8], so far, P2P systems have occupied more than 60% of Internet traffic in China. We can see that detection time, accuracy and overhead all have significant effects on the improvement of P2P system performance. On the other hand, P2P systems may contain participants from any corner of the Internet, different capabilities of processing and network accessing generate complex network environment [9]. For example, comparing a task completed in open network with the one that is completed on a company's internal network, huge difference exists on their network conditions. Therefore, the study of adaptive failure detectors that can ensure the QoS of failure detection with lower overhead according to the changes in network conditions is very significant for building P2P applications.

Most of the current study on QoS-based adaptive failure detector is based on the classic adaptive model proposed by Chen [10], a series of adaptive detectors have been proposed to achieve the quantitative QoS metrics on the dynamic adjustment of detector parameters under different network conditions [10–12]. However, P2P applications covers the entire Internet, complex network conditions and the rapid joining and leaving by large number of nodes make the message loss rate very high, which may easily lead to an increase in false positive rate [13]. Therefore, baseline detection strategy based on retransmission mechanism [4,14] has been widely employed in P2P networks to reduce the impact of message loss on detection results and improve detection accuracy. However, the QoS of baseline strategy cannot be evaluated by Chen's classic model. To address the problem, this paper establishes a novel QoS evaluation model for the baseline detection strategy. The relationship between the detection period and the QoS is discussed and on this basis, a QoS-based adaptive failure detector (B-AFD) is proposed. Under the quantitative control by QoS basic metric $\left(T_{D}^{U},T_{MR}^{L},T_{M}^{U}\right)$ [10], B-AFD can adapt to the changing network environment and achieves better accuracy and detection time with lower detection loads. Meanwhile, experimental analysis shows that B-AFD has better adaptability to P2P networks as compared to the traditional baseline strategy and the adaptive detector based on Chen's model.

## 2. Related Work

### 2.1. QoS Metrics for Failure Detector

It is well known that in the asynchronous distributed systems with crashed nodes, many important and fundamental problems (e.g., consensus) cannot be solved due to the fact that crashed nodes cannot be distinguished correctly from the nodes that response slowly [15]. Failure detector was first proposed and formally specified by Chandra [16] as an effective way to enhance the computational model for asynchronous system. Currently it has been applied widely in many related fields such as grid computing, cluster management, P2P networks, *etc.* [17,18]. To evaluate the ability of failure detector to solve consensus problems, Chandra categorized the detection capabilities according to completeness and accuracy. However, in actual systems, an application is much concerned about how fast a failure detector detects crashes and how well it avoids false detections, that is, the detection time and accuracy of failure detector. To evaluate these attributes accurately and quantitatively, Chen *et al.* proposed a set of quantitative QoS metrics [10,12]. Let *T* denote the output of failure detector when the monitored node is normal and *S* denotes the output when the node is failed. *T*-transition occurs when the output changes from *S* to *T*, while *S*-transition occurs when the output changes from *T* to *S*. The QoS provided by failure detector can be quantitatively described by the following basic metrics [10].

Detection time (*T_D_*): *T_D_* measures the time that elapses from the moment when a node crashes to the time when it starts being suspected (*S*-transition).

Mistake recurrence time (*T_MR_*): *T_MR_* measures the time between two consecutive mistakes, it is a random variable representing the time that elapses from an *S*-transition to the next one.

Mistake duration (*T_M_*): *T_M_* measures the time it takes the failure detector to correct a mistake, it is a random variable representing the time that elapses from an *S*-transition to the next *T*-transition. *T_MR_* and *T_M_* can specify the detection accuracy.

Based on these three primary metrics, other QoS metrics can be uniquely derived, such as average mistake rate (λ*_M_*) and query accuracy probability (*P_A_*), *etc.*

### 2.2. Adaptive Failure Detector

QoS requirement is the main basis for the design of failure detector. Most of failure detectors presented in the literature are implemented using the timeout-based mechanism [19], in which the probing messages are sent out periodically to detect the states of other nodes. Under this mechanism, a detector's behavior can be determined by the failure detection period η and the timeout value δ. As the network conditions (e.g., packets loss rate, transmission delay, *etc.*) are changing constantly, the QoS of failure detection cannot be guaranteed to meet the design requirements all the time. Therefore, adaptive failure detection algorithms have been proposed to adapt to the changing network conditions by adjusting the parameters η and δ automatically. Initially, such adjustments were achieved by modifying δ according to the prediction of the arrival time for detection messages and a simple trade-off was made between detection time and accuracy. Falai [20] presented the performance comparison of various commonly used prediction methods such as LAST, MEAN, linear sequence *etc.* After proposing the QoS metrics, Chen [9] presented a classic adaptive model based on the network probability model, and on the basis, many adaptive failure detectors [10–12] are proposed to achieve the quantitative QoS metrics required by upper application with lower detection overhead under different network conditions. However, to reduce the impact on detection accuracy by the high message loss rate resulted from complexity of network and high churn, baseline detection strategy based on retransmission mechanism has been employed widely in P2P systems [4,7,14]. These changes make Chen's classic adaptive model no longer applicable to P2P systems. In order to provide the service of QoS-based adaptive failure detection in P2P systems, this paper establishes a novel QoS evaluation model for the baseline detection strategy that is based on retransmission mechanism and PULL style. Furthermore, a QoS-based adaptive failure detector B-AFD is proposed for P2P networks.

## 3. QoS Evaluation Model

### 3.1. Baseline Failure Detection Strategy

**Figure 1.**
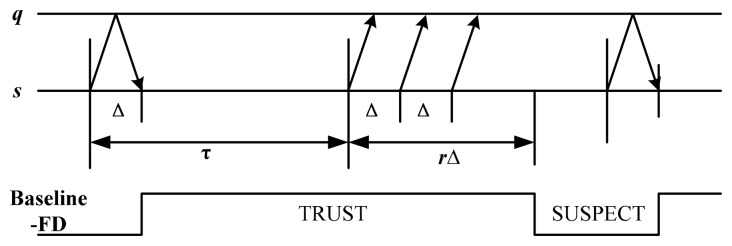
Baseline detection strategy.

We consider an asynchronous distributed systems which consists of a set of *n* nodes. The same failure model is employed here as crashing used by Chandra [16]. The channels between nodes are fair-lossy link [21], that is, only a finite number of messages are allowed to be lost, assume the message loss probability is *p_l_*. To simply the description, we consider a failure detector module in node *s*, which detects the state of node *q*. PULL style [22] is used in the baseline detection strategy and retransmission mechanism is designed in each detection period as shown in Figure 1.

In Figure 1, failure detector module of *s* sends a probing message “are you alive” to the monitored node *q* in each detection period (denoted as τ). Node *q* will send an “ack” message back to acknowledge the receipt of the probing message. If *s* receives an acknowledge message from *q* in interval Δ, then the detector output is *T*. Otherwise, probing packets are sent out periodically at interval Δ. If *r* consecutive packets cannot receive the “ack” message, then the output of detector is *S*.

### 3.2. QoS Analysis

The analysis of QoS evaluation metrics is an important basis for the study of QoS-based adaptive failure detector. Theorem 1 gives the QoS calculation model based on the baseline detection strategy shown in Figure 1.

#### Theorem 1

*In the baseline detection strategy shown in Figure 1, let p_l_ denote the message loss probability, P*(*D* < *x*) *denote the probability distribution of detection message delay* (*D*) *and s denote the number of bytes in detection message. We have:*

(1) *Average mistake recurrence time* $E(T_{MR})=\frac{\tau }{p^{r}(1-p^{r})}$
(2) *Average mistake duration* $E(T_{M})=\frac{\tau }{1-p^{r}}-\frac{r\Delta }{1-p^{r}}+\frac{\Delta }{1-p}$
(3) *Detection time T_D_* ≤ τ + *r*Δ
(4) *Average detection overhead* $E(B)=\frac{1-p^{r}}{1-p}\cdot \frac{s}{\tau }$ *where p* = *p_l_* + (1 − *p_l_*) *P*(*D* > Δ).

*The detection message delay is the time that elapses from the sending of probing message to the receipt of “ack” message. We suppose the monitored node q always keeps accurate. For any detection message mq_ij_(i* > 0, *r* ≥ *j* > 0*), the probability that no “ack” is received at time* (*i*τ + *j*Δ) *is p_l_* + (1 − *p_l_*) *P*(*D* > Δ)*, that is, the value of p in Theorem 1. To complete the proof of Theorem 1, two lemmas should be given first*.

#### Lemma 1

$E(T_{MR})=\frac{\tau }{p^{r}(1-p^{r})}$, *where p* = *p_l_* + (1 − *p_l_*) *P*(*D* > Δ).

**Figure 2.**
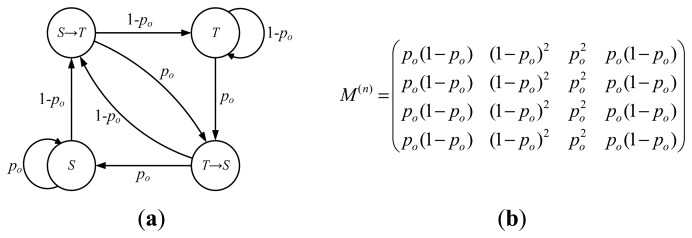
(**a**) The state transition diagram; (**b**) n-step transition probability matrix (n > 2).

*Let* {*X_i_, i* > 0} *be a random sequence, where random variable X_i_represents the output state of the detector at time* (*i*τ + *r*Δ). *For the baseline strategy in* *Figure 1*, *the state space can be defined as G* = (*T* → *S, S* → *T, S, T*)*, e.g., S-transition, T-transition, maintaining S state and maintaining T state. The state transition diagram and the n-step transition probability matrix M^(n)^(n > 2) for* {*X_i_*, *i* > 0} *are shown in* *Figure 2*, *where p_0_is the probability that none of the detection messages is received during a detection period. Thus, we have p*_0_ = *p^r^*.

*From the Figure 2, we can see that the value of variable X_i_* (*i* > 0) *is determined jointly by* {*mq_ij_*, 0 ≤ *j* ≤ *r* − 1} *and the value of X_i-1_. Moreover, it is irrelevant to X_j_* (0 < *j* < *i* −1)*, that is, P*(*X_i_* | *X_i_*_−1_) = *P*(*X_i_* | *X_i_*_−1_, *X_i_*_−2_, …*, X*_0_). *Hence, the random sequence* {*X_i_*, *i* > 0} *is a finite state Markov chain. Since 0 < p_0_< 1, we can get that* {*X_i_*, *i* > 0} *is a recurrent Markov chain and the state T* → *S is ergodic from the transition matrix M^(n)^. For state T* → *S, there exists* $\underset{n\to \infty }{\lim}M_{T\to S,T\to S}^{\left(n\right)}=p_{o}(1-p_{o})>0$, *the average recurrence time is* 1/*p*_0_(1 − *p*_0_). *Consider the time needed for an occurrence of state transition in a detector system as* τ*, we have E*(*T_MR_*) = τ/*p^r^* (1 − *p^r^*).

#### Lemma 2

*In the baseline detection strategy shown in Figure 1, the query accuracy probability is* $P_{A}=(1-p^{r})+\frac{r\Delta }{\tau }p^{r}-\frac{\Delta }{\tau }p^{r}\frac{1-p^{r}}{1-p}$, *p* = *p_l_* + (1 − *p_l_*) *P*(*D* > Δ).

#### Proof of Lemma 2

Query accuracy probability *P_A_* is the probability that the failure detector's output is *T* at a random time *t*. That is, the probability that users get an accurate output when query the failure detector at any time.

Consider any detection period *i* (*i* > 0) in baseline strategy, suppose *Y_i_* is a random variable representing the time that the detector output is *T* during the period [*i*τ, (*i* + 1)τ]. Let's discuss *Y_i_* under two situations:

**The final output is T during the detection period** [(*i* − 1) τ, *i*τ)

In this case, in detection period [*i*τ, (*i* + 1)τ), if at least one of the *r* detection messages have been acknowledged in interval Δ, then the output will keep *T* during the entire period τ. Otherwise, *S*-transition will occur at time (*i*τ + *r*Δ). Hence, we have *E*(*Y_i_*)*_T_* = *r*Δ · *p^r^* + τ ·(1 − *p^r^*).

**The final output is S during the detection period** [(*i* − 1) τ, *i*τ)

If the detector's state is *S* in detection period [(*i* − 1) τ, *i*τ), then the moment *T*-transition occurs in period *i* depends on the time when the first detection message is acknowledged. Thus, we have

$$\begin{array}{cc} E{\left(Y_{i}\right)}_{S} & =\overset{r-1}{\underset{i=0}{\text{∑}}}\left(1-p)p^{i}(\tau -(i+1)\Delta \right)\\ & =\tau (1-p^{r})-\Delta \frac{1-p^{r}}{1-p}+r\Delta p^{r} \end{array}$$

In summary, the time that the detector's output is *T* during the period [*i*τ, (*i* + 1)τ) is

$$\begin{array}{ll} E(Y_{i}) & =(1-p^{r})E{\left(Y_{i}\right)}_{T}+p^{r}E{\left(Y_{i}\right)}_{S}\\ & =\tau (1-p^{r})+r\Delta p^{r}-\frac{1-p^{r}}{1-p}p^{r}\Delta \end{array}$$

In the above equation, the value of *E*(*Y_i_*) is irrelevant to the detection period *i*. Hence, the query accuracy probability is

$$P_{A}=(1-p^{r})+\frac{r\Delta }{\tau }p^{r}-\frac{\Delta }{\tau }p^{r}\frac{1-p^{r}}{1-p}$$

Now we prove Theorem 1 using Lemma 1 and Lemma 2.

#### Proof of Theorem 1

(1) It has been proved by Lemma 1.
(2) According to Chen's QoS metrics, we have *P_A_* = 1 − *E*(*T_M_*)/*E*(*T_MR_*). Then we get *E*(*T_M_*) = (1 − *P_A_*)*E*(*T_MR_*). According to Lemma 2, we have
  $$E(T_{M})=\frac{\tau }{1-p^{r}}-\frac{r\Delta }{1-p^{r}}+\frac{\Delta }{1-p}$$
(3) In the detection period *i*, if node *q* crashes during [*i*τ, (*i* + 1)τ), then the failure will be detected no later than ((*i* +1τ + *r*Δ)). Thus, the failure detection time satisfies *T_D_* ≤ τ + *r*Δ.
(4) In every detection period of baseline strategy, at most *r* messages are sent out and no more messages will be sent after the first acknowledgement message is received. In detection period *i*, suppose there is a random variable *Z_ij_*,
  $$Z_{ij}=\{\begin{array}{l} \begin{array}{cc} 1 & \text{if}\;mq_{ij}\;\text{has been acknowledged by time}\;(i\tau +j\Delta \end{array})\\ \begin{array}{cc} 0 & \text{else} \end{array} \end{array},i>0,r\geq j>0$$

We have *P*(*Z_ij_* = 1) = 1 − *p* and *Z_ij_* satisfies the Bernoulli distribution with success probability as 1 − *p*. Hence, the number of messages *N_m_* sent within a detection period satisfies the geometric distribution that is

$$E(N_{m})=\overset{r}{\underset{j=1}{\text{∑}}}p^{j-1}(1-p)\cdot j+rp^{r}$$

Now we can get the average detection loads of the algorithm as $E(B)=\frac{1-p^{r}}{1-p}\cdot \frac{s}{\tau }$.

In summary, Theorem 1 is proved.

### 4. QoS-Based Adaptive Failure Detector B-AFD

$\left(T_{D}^{U},T_{MR}^{L},T_{M}^{U}\right)$ is used to describe the quantitative requirements of system designers on the detection accuracy and time of failure detectors, where $T_{D}^{U}$ is an upper bound on the detection time, $T_{MR}^{L}$ is a lower bound on the average mistake recurrence time, and $T_{M}^{U}$ is an upper bound on the average mistake duration. B-AFD is based on baseline failure detection strategy, where parameters *r* and τ are adjusted automatically to adapt to the dynamic network environment in P2P system so that the requirements of QoS metrics $\left(T_{D}^{U},T_{MR}^{L},T_{M}^{U}\right)$ are met with relatively low detection overhead. The B-AFD failure detector is described in Alogorithm 1.



**Alogrithm 1:** B-AFD failure detector
For node *s*:detector_module: at time τ*_i_*: (the *i*th detection period)  (*r_b_*, τ_*b*_)=get_opti_para( );  if ((*r_b_* = 0 ∧ τ_b_ = 0)) then      exit (“QoS cannot be achieved”);    τ*_i_*_+1_ = τ*_i_* + τ*_b_*; *j*←0;    do {     send *mq_ij_* to node *q* at timeτ*_i_* + *j*Δ;     if (receive *ma_ij_* beforeτ*_i_* + (*j* + 1)Δ) then      result ← *T*; break;     else *j*++;    } while(*j* < *r_b_*);    if (*j* ≥ *r_b_*) then result ← *S*;gatherer_module:  upon receive *ma_ij_* from *q* do   add(*t_current_*-(τ*_i_* + *j*Δ)) to *W_D_*;For node *q*:  upon receive *mq_ij_* form *s* do   send *ma_ij_* to *s*;


B-AFD failure detector is composed of detector_module and gatherer_module. In detector_module, at most *r_b_* probing messages *mq_ij_* are sent out during each detection period. If none of the messages can receive the corresponding “ack” message *ma_ij_* successfully, the detector outputs *S* for node *q*; otherwise, the detector believes that *q* maintains accurate state. From Alogorithm 1, we can see that function get opti para is the core mechanism in B-AFD for the automatic adaptation to network states, which is called to compute the parameters *r* and τ at the beginning of each detection period. As shown in Alogorithm 2, the algorithm get opti para uses $\left(T_{D}^{U},T_{MR}^{L},T_{M}^{U}\right)$ as input parameters so that the detector can be configured to meet the QoS required by upper applications with the minimum overhead. The gatherer_module in B-AFD creates a sliding window *W_D_* with fixed size (*w*). The detection message delay of the *w* probing messages that are recently acknowledged is saved to calculate the message loss probability *p_l_* and establish samples for the estimation of the probability distribution of detection message delay *P*(*D* < *x*).



**Alogorithm 2:** Adaptive configuration of parameters
get_opti_para( )Input: 
$T_{D}^{U},T_{MR}^{L},T_{M}^{U}$Output: *r*,τStep 1: compute *p* = *p_l_* + (1 − *p_l_*) *P*(*D* > Δ);Step 2: if 
$T_{M}^{U}<\Delta /(1-p)$ then return (0,0);Step 3: compute 
$r_{\max}=\left\lfloor T_{D}^{U}/2\Delta \right\rfloor$, *R* = [1, *r*_max_];   compute *R_L_* = {*r*|*L*(*r*) ≥ 0, *r* ∈ *R*} and *R_U_* = {*r*|*U*(*r*) ≥ 0, *r* ∈ *R*};   
$L(r)=(1-p^{r})(T_{M}^{U}-\frac{\Delta }{1-p}-T_{MR}^{L}p^{r})+r\Delta$   
$U(r)=T_{D}^{U}-T_{MR}^{L}p^{r}(1-p^{r})-r\Delta$Step 4: if *R_L_* ∩ *R_U_* ==Ø then return (0,0);Step 5: Compute *Output* = {(*r*, τ) | *r* ∈ *R_L_* ∩ *R_U_ and* τ *satisfies* (1)};Step 6: find (*r_b_*, τ_b_) ∈ *output* such that *B*(*r_b_*, τ*_b_*) = min{*B*(*r*,τ) | (*r*, τ) ∈ *output*};   
$B(r,\tau )=\frac{1-p^{r}}{1-p}\cdot \frac{s}{\tau }$Step 7: return (*r_b_*, τ*_b_*)


### Theorem 2

*If the return value of the algorithm get_opti_para in* Alogorithm 2 *satisfies* (*r* > 0) ∧ (τ > 0)*, then the outputs of the failure detector B-AFD can meet the QoS requirements* $\left(T_{D}^{U},T_{MR}^{L},T_{M}^{U}\right)$, *else B-AFD cannot achieve the given QoS requirements*.

### Proof of Lemma 2

According to the results in Theorem 1, there are $E(T_{MR})\geq T_{MR}^{L}$, $E(T_{M})\leq T_{M}^{U}$ and $T_{D}\leq T_{D}^{U}$. The parameters *r* and τ satisfy the constraint relationship in Equation (1).

(1) $$\left\{ \begin{array}{l} \tau \leq T_{M}^{U}(1-p^{r})+r\Delta -\frac{1-p^{r}}{1-p}\Delta \\ \tau \geq T_{MR}^{L}p^{r}(1-p^{r})\\ \tau \leq T_{D}^{U}-r\Delta \\ \tau \geq r\Delta \end{array}\right.$$

According to Equation (1), if τ exists, then it need satisfy

(2) $$\{\begin{array}{ll} (1-p^{r})(T_{M}^{U}-T_{MR}^{L}p^{r}-\Delta /(1-p))+r\Delta \geq 0 & \left(a\right)\\ T_{D}^{U}-T_{MR}^{L}p^{r}(1-p^{r})-r\Delta \geq 0 & \left(b\right)\\ T_{M}^{U}-\Delta /(1-p)\geq 0 & \left(c\right)\\ T_{D}^{U}-2r\Delta \geq 0 & \left(d\right) \end{array}$$

From Equation (2-*c*), we get $T_{M}^{U}<\Delta /(1-p)$ (step2), otherwise the detector is unable to meet the given QoS requirements. From Equation (2-*d*) we have $r\leq T_{D}^{U}/2\Delta$, then the range of *r* satisfies $1\leq r\leq T_{D}^{U}/2\Delta$. Meanwhile, it can be proved that on interval $\left[1,T_{D}^{U}/2\Delta \right]$, *L*(*r*) is monotonically increasing and *U*(*r*)is monotonically decreasing. According to the constraints Equation (2-*a*,2-*b*), we can get the upper bound *r_U_* and the lower bound *r_L_* for parameter *r* respectively. If *r_L_* ≤ *r_U_*, that is, the condition *R_L_* ∩ *R_U_* ≠ Ø is satisfied in step 4, then *r* ex0ists; otherwise the detector cannot meet the given QoS requirements under current network conditions. In step 5, using Equation (1), the collection *Output* contains all the combinations of values for (*r*, τ) which are capable of meeting the QoS requirements. By the screening procedure in step 6, we can get the parameter configuration (*r_b_*, τ*_b_*) which achieves the minimum detection overhead. Therefore, if the return value of get opti para is non-zero, then the outputs of the failure detector B-AFD configured according to that return value will be able to meet the QoS requirements $\left(T_{D}^{U},T_{MR}^{L},T_{M}^{U}\right)$. Theorem 2 is proved.

### 5. Experimental Results and Analysis

To evaluate the performance of B-AFD, we compare B-AFD with two typical failure detectors. One is traditional baseline detection strategy with fixed parameters (shown in Figure 1), which has been used the most commonly in current P2P environment. This experiment is to verify the improvement on detection accuracy and overhead by the adaptive mechanism in B-AFD. The other one is QoS-based adaptive detector NFD-E [10], which is compared to verify B-AFD's ability of adapting to the complex P2P network environment. To ensure the authenticity of the experiments, some nodes in currently prevalent P2P applications (emule and Bittorrent) are selected as detection objects, the failure detector node located in Harbin City (China).

In P2P systems, nodes may come from any corner of the Internet with huge difference in network conditions, thereby, the experiments are carried out on two sets of nodes which represent two typical network conditions in P2P networks. One group (dataset 1) contains monitored nodes located in China, which have good network connections with detector node, the message delay and message loss probability are low (*p_l_* = 0.39%, *E*(*D*) = 125 ms, where *E*(*D*) is the expectation of detection message delay). The other group (dataset 2) mainly consists of monitored nodes located in the United States, which have relatively poor connections with detector node (in China), in which the message transmission delay and loss rates are high (*p_l_* = 3.65%, *E*(*D*) = 412 ms). We choose exponential distribution for detection message delay D with reference to Lakshman's research [23] about failure detector in a P2P storage system, *i.e.*, *P*(*D* ≤ *x*) = 1 − *e*^−^*^x^*^/^*^E(D)^*, for all *x* > 0.

#### 5.1. Comparisons with the Traditional Baseline Strategy

**Figure 3.**
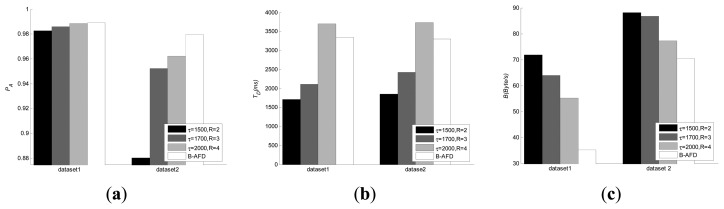
Comparison with baseline strategy. (**a**) detection accuracy; (**b**) detection time; (**c**) overhead.

The comparison with baseline strategy is evaluated in three aspects: Detection accuracy, detection time and overhead. Since baseline uses fixed parameters, for fairness, three sets of different parameters are selected for baseline under different network environment. The comparison results are shown in Figure 3.

As we can see from Figure 3, under good network conditions (dataset 1), all the four sets of experiments have achieved high accuracy. Smaller detection period leads to lower detection time, but with higher detection overhead. In the experiments on dataset 2, where the distance of the nodes is far and network condition is poor, the detection accuracy drops significantly for the baseline with smaller detection period. The changes in network conditions make huge difference on QoS, therefore, baseline strategy with fixed period is not applicable to the kind of large-scale distributed systems, such as P2P. Experimental results have shown that B-AFD detectors have good adaptability to different network environment due to the detection parameters adjusted adaptively according to network changing, there is little change in the accuracy and detection time under different network environment and the specified QoS metrics are still satisfied while minimum overhead are maintained. Moreover, Figure 3-c shows that the decrease in overhead is more obvious when the network condition is better.

#### 5.2. Comparisons with NFD-E Adaptive Detector

**Figure 4.**
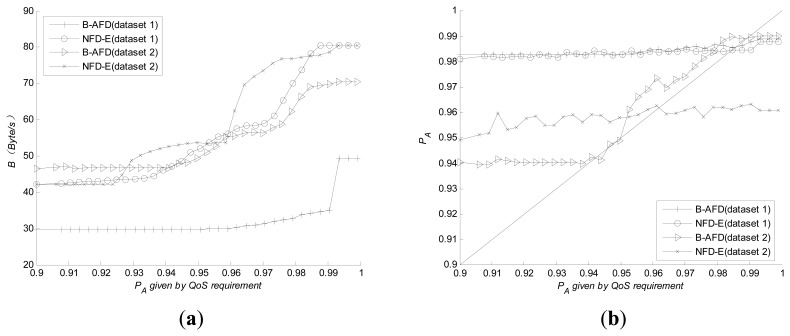
Comparisons with NFD-E. (**a**) Detection overhead (**b**) adaptability.

NFD-E is a classical adaptive failure detector proposed by Chen [8], which can meet the requirements of quantitative QoS metrics under different network environment as well without retransmission mechanism. Therefore, experiments are carried out to compare B-AFD and NFD-E in two aspects: The overhead needed to meet the same QoS metrics and the capability of adapting to complex network. The results are shown in Figure 4.

To obtain the same detection accuracy, Figure 4a shows that the detection overhead generated by B-AFD is significantly lower than NFD-E, especially when the requirement of detection accuracy is higher. As can be seen from Figure 4b, B-AFD demonstrates better adaptability under poor network conditions. Given the same QoS metrics, from the experimental results of dataset 2, NFD-E is no longer meet the requirement when the query accuracy exceeds 96%. However, nearly 99% of the query accuracy requirements are met by B-AFD under the same network environment. It is clear that the retransmission mechanism can significantly improve the accuracy under poor network conditions without adding overhead. Under good network conditions, B-AFD demonstrates a very close adaptability to NFD-E while keeping obvious advantage in detection overhead as shown in Figure 4. Therefore, B-AFD is more appropriate for the kind of large-scale distributed systems, such as P2P that have wide coverage and complex network conditions, especially for the structured P2P systems whose node routing tables may contain the nodes from internal LAN and the nodes from overseas at the same time.

### 6. Conclusions

P2P networks have become the major source of Internet traffic. The study of adaptive failure detector with low detection overhead provides an important method to reduce the overhead of P2P networks. To address the challenges posed to failure detection by the complexity of network and high churn in P2P system, this paper establishes a novel QoS evaluation model for the baseline detection strategy based on retransmission mechanism. On this basis, an adaptive failure detector B-AFD is proposed based on the basic QoS metrics $\left(T_{D}^{U},T_{MR}^{L},T_{M}^{U}\right)$. It can adapt to the changing network environment and achieve better detection accuracy and time with lower overhead. Meanwhile, experimental analysis shows that compared to Chen's adaptive detectors, B-AFD achieves better adaptability to the complex network conditions in P2P systems.

## References

[1] Eng K.L., Crowcroft J., Pias M., Sharma R., Lim S. (2005). A survey and comparison of peer-to-peer overlay network schemes. IEEE Commun. Surv. Tutor..

[2] Kurian J., Sarac K. (2012). A survey on the design, applications, and enhancements of application-layer overlay networks. Acm Comput. Surv..

[3] Xiong N., Vasilakos A.V., Wu J., Yang Y.R., Rindos A., Zhou Y.Z., Song W.Z., Pan Y. A Self-tuning Failure Detection Scheme for Cloud Computing Service.

[4] Zhuang S.Q., Geels D., Stoica I., Katz R.H. On failure detection algorithms in overlay networks.

[5] Ohzahata S., Kawashima K. (2011). An experimental study of peer behavior in a pure P2P network. J. Syst. Softw..

[6] Benhamida F.Z., Challal Y., Koudil M. ALLONE: A New Adaptive Failure Detector Model for Low-Power Lossy Networks.

[7] Castro M., Costa M., Rowstron A. Performance and dependability of structured peer-to-peer overlays.

[8] Dedinski I., Hofmann A., Sick B. Cooperative keep-alives: An efficient outage detection algorithm for p2p overlay networks.

[9] Tian J., Dai Y. Understanding the dynamic of peer-to-peer systems.

[10] Chen W., Toueg S., Aguilera M.K. (2000). On the quality of service of failure detectors. IEEE Trans. Comput..

[11] Bertier M., Marin O., Sens P. Implementation and performance evaluation of an adaptable failure detector.

[12] Tiejun M., Hillston J., Anderson S. (2010). On the quality of service of crash-recovery failure detectors. IEEE Trans. Dependable Secur. Comput..

[13] Stutzbach D., Rejaie R. Understanding churn in peer-to-peer networks.

[14] Price R., Tino P. Still alive: Extending keep-alive intervals in P2P overlay networks.

[15] Fischer M.J., Lynch N.A., Paterson M.S. (1985). Impossibility of distributed consensus with one faulty process. J. ACM.

[16] Chandra T.D., Toueg S. (1996). Unreliable failure detectors for reliable distributed systems. J. ACM.

[17] Lavinia A., Dobre C., Pop F., Cristea V. A failure detection system for large scale distributed systems.

[18] Costache S., Ropars T., Morin C. (2010). Towards highly available and self-healing grid services.

[19] Pasin M., Fontaine S., Bouchenak S. Failure detection in large scale systems: A survey.

[20] Falai L., Bondavalli A. Experimental evaluation of the QoS of failure detectors on wide area network.

[21] Zhang J., Chen W. (2009). Implementing uniform reliable broadcast with binary consensus in systems with fair-lossy links. Inf. Process. Lett..

[22] Felber P., Defago X., Guerraoui R., Oser P. Failure detectors as first class objects.

[23] Lakshman A., Malik P. (2010). Cassandra: A decentralized structured storage system. ACM SIGOPS Oper. Syst. Rev..

